# The duration of *Plasmodium falciparum* infections

**DOI:** 10.1186/1475-2875-13-500

**Published:** 2014-12-16

**Authors:** Elizabeth A Ashley, Nicholas J White

**Affiliations:** Mahidol-Oxford Tropical Medicine Research Unit (MORU), Faculty of Tropical Medicine, Mahidol University, Bangkok, Thailand; Centre for Tropical Medicine and Global Health, Nuffield Department of Medicine, University of Oxford, Oxford, UK

**Keywords:** *Plasmodium falciparum*, Transfusion malaria, Longevity, Asymptomatic, Sub-microscopic

## Abstract

*Plasmodium vivax* and *Plasmodium ovale* are often considered the malaria parasites best adapted to long-term survival in the human host because of their latent exo-erythrocytic forms. The prevailing opinion until the middle of the last century was that the maximum duration of *Plasmodium falciparum* infections was less than two years. Case reports and series investigating blood donors following accidental malaria infection of blood transfusion recipients and other sporadic malaria cases in non-endemic countries have shown clearly that asymptomatic *P. falciparum* infections may persist for up to a decade or longer (maximum confirmed 13 years). Current policies in malaria-free countries of excluding blood donors who have lived in malarious areas are justified. Vigilance for longer than three years after declaring elimination in an area may be needed.

## Background

*Plasmodium vivax* and *Plasmodium ovale* are often considered the malaria parasites best adapted to long-term survival in the human host because of their latent exo-erythrocytic forms. Yet *Plasmodium malariae,* which has no latent forms, can persist asymptomatically in the blood for decades, and perhaps throughout the entire life of the host [1]. Many of these persistent asymptomatic *P. malariae* infections have been revealed by blood donation [2], with illness in the recipient (but not the donor). The maximum duration of asymptomatic infection is relevant both to transfusion risks and also in planning and evaluating malaria elimination.

Characterization of the natural history of untreated or partially treated *Plasmodium falciparum* malaria comes first from the early descriptions of neurosyphilis patients who were given malaria. Malariatherapy, the brainchild of the Viennese neuropsychiatrist Julius Wagner-Jauregg, was introduced as a treatment for neurosyphilis in the 1920s, and was widely used until supplanted by penicillin in the 1950s. The malariatherapy parasite of choice was generally *P. vivax* rather than the more dangerous *P. falciparum*, but all species were used. Detailed studies of syphilis patients infected with strains of *P. falciparum* reported durations of infection between 50 and 500 days depending on the infecting strain [3, 4].

The duration of *P. falciparum* infections was debated by malariologists during the first era of malaria eradication (in the 1950s) as it was a key consideration in the investigation of any sporadic cases in the post-elimination period. A review by Covell in 1960 presented the prevailing opinion that *P. falciparum* infections generally did not continue beyond one year although he noted nine cases where infection had recurred between one and two years after the primary infection, and he cited Ciuca’s review of 12,842 cases of experimental malaria from Romania showing a maximum persistence of *P. falciparum* infections of 27 months [5–7]. In 1964 Jacques Verdrager, a WHO malariologist, reviewed the literature again and added his own observations from the Malaria Eradication Project in Mauritius which suggested that *P. falciparum* could persist in humans for at least three years. One example was probable transfusion malaria from a donor who must have been parasitaemic for more than two years. Another was a presumed recrudescence of falciparum malaria in a young woman who had a documented episode of malaria three years previously, after which she had resided in an area considered free of malaria transmission [8]. Cohort studies in endemic areas have also proved informative. Between 1931 and 1934 in Puerto Rico, Earle followed a cohort of 71 schoolchildren prospectively with very frequent blood sampling for microscopy and found that some *P. falciparum* infections continued for up to 121 weeks [9].

A mathematical modelling approach has been used to estimate the duration of falciparum infections using repeated cross-sectional survey data from other cohorts, such as the subjects in the Garki project in the 1970s. Estimates for the total duration of infection across all age groups were long at 602 days (95% confidence interval (CI) 581–625) for Pare-Taveta (in Kenya/Tanzania), 734 days (95% CI 645–849) for West Papua, and 1,329 days (95% CI 1,193-1,499) for Garki in Nigeria [10]. Until molecular genotyping was introduced it was not possible to distinguish chronic single infections from multiple repeated infections in endemic areas, suggesting that the predicted longevity of blood stage infections based on microscopy data might be overestimated. The protracted duration of natural infections was demonstrated in Eastern Sudan where 16 of 43 individuals who had either had a malaria illness during the transmission season or were PCR positive for *P. falciparum* malaria at the end of the malaria season remained chronically infected throughout the dry season and the following rainy season with the same genotype [11].

In high-transmission areas asymptomatic parasitaemia is very common. The ability of individuals to control their parasitaemia at low levels without symptoms (premunition) results from the gradual acquisition of immunity following repeated or protracted exposure. In these areas uncomplicated and severe malaria are predominantly diseases of young children. Superinfection is usual with individuals harbouring multiple, genetically unrelated, parasite clones in a constantly changing equilibrium, often generating transmissible densities of gametocytes but remaining below the combined asexual parasite density that provokes illness [12–14].

### Hyperreactive malarial splenomegaly

Sometimes the host immune response to repeated malaria infection is excessive, manifest by the syndrome of hyper-reactive malarial splenomegaly (HMS); marked splenomegaly with hypersplenism and hypergammaglobulinaemia (in particular raised IgM). *Plasmodium falciparum* is usually responsible. Anti-malarial antibody titres are high but microscopy examination of blood smears is usually negative. Persistent or recurrent active malaria infection is confirmed by response (shrinkage of the spleen) to effective anti-malarial treatment (often in a prolonged course to prevent re-infection). Typical histopathological findings in the spleen are non-specific cellular hyperplasia and lymphocytic infiltration without malaria pigment. Two cases of symptomatic falciparum malaria occurring post splenectomy (performed for suspected lymphoma) were reported from France. Both were in immigrants who had not visited a malaria-endemic area for at least two years. Their diagnoses were changed to HMS retrospectively and indicate persistent active infection throughout this more than two years interval [15]. Similarly, a case report in Australia described a 28-years-old woman, born and brought up in Eritrea and Sudan, who developed falciparum malaria following splenectomy nine years after arriving in Australia. Histopathological findings in the spleen were compatible with HMS [16]. Examples of chronic falciparum infection, such as these from non-endemic countries, are very informative since the possibility of re-infection can be excluded with confidence. The richest source of information on persistent malaria comes from reports of malaria in recipients of blood transfusions or transplants in non-endemic areas.

### Transfusion- and transplant-transmitted malaria

The first report of malaria as an accidental consequence of an artery to vein blood transfusion was described in 1910 by Woolsey [17]. The efficiency of blood transfusion in transmitting low density parasitaemias to naïve recipients was exploited by Fairley in his classic studies of the biology and chemotherapy of human malaria conducted in Cairns in the 1940s [18]. Fairley also observed protracted infections in some of his volunteers. A review of transfusion malaria published in 1974 summarized 2,001 cases of transfusion-transmitted malaria between 1911 and 1972, of which at least 88 were due to *P. falciparum* [19]. The details of most of these cases are not in the public domain. In those for whom information was available, the mean time between the transfusion and the onset of symptoms of malaria was 10.5 days (i.e., five asexual cycles) for the *P. falciparum* cases. Assuming a multiplication factor of approximately ten-fold per cycle and a pyrogenic threshold of 10^8^ parasites in total in an adult, this suggests an inoculum of ~1,000 parasites in a blood bag or bottle of approximately 500 mL. Thus the likely donor density approximated an average of 2 parasites/mL, well below the pyrogenic (and microscopy detection threshold) density of ≥50,000/mL.

## Review

A search of Pubmed and Embase was performed for case reports of transfusion or transplant transmitted falciparum malaria or for cases of falciparum malaria presenting more than six months after leaving an endemic area. Cited references were used to identify other cases. Cases of transfusion-transmitted malaria from the USA were searched for in the annual MMWR malaria surveillance reports. Searches were also performed in websites of blood banks and the international haemovigilance network [20]. Reports of cases were excluded if the species was described as ‘tertian’ malaria without further clarification, if in languages other than English or French, or if the probable duration of the infection in the case or donor was six months or less.

**Figure 1:**
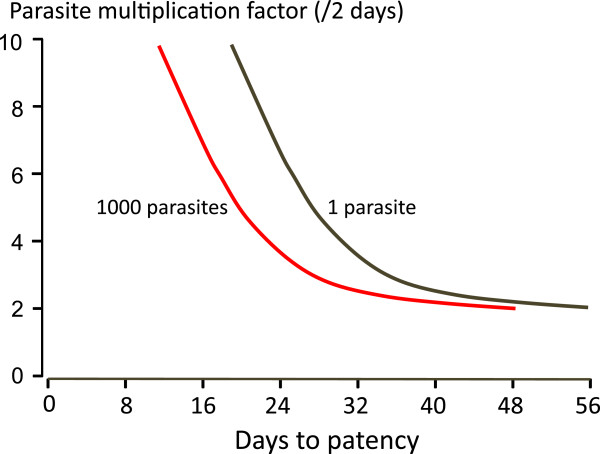
**Relationship between size of parasite inoculum and time to microscopy detection.**

**Figure 2:**
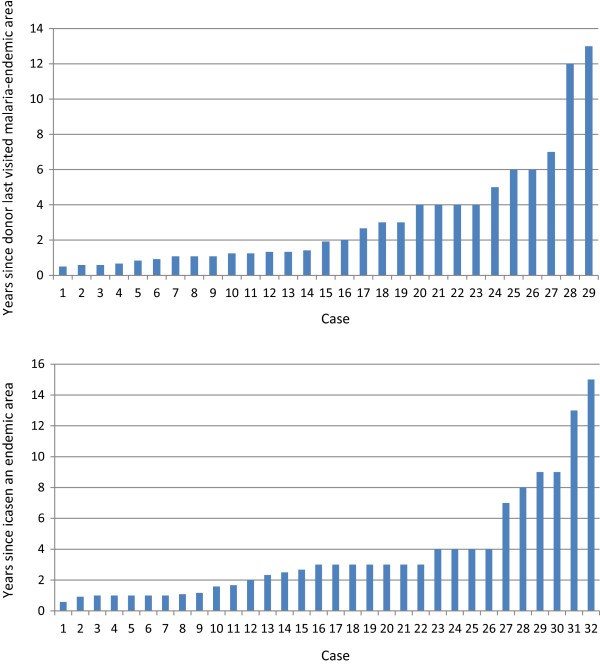
**Cases of transfusion or late-presenting clinical malaria showing minimum interval since last malaria exposure of donor or case. A** Transfusion-transmitted falciparum malaria **B** Non-transfusion cases.

Twenty-nine cases of transfusion and transplant-transmitted falciparum malaria are summarized in Table 1. The time interval from last possible exposure to malaria and the accidental transmission of the infection ranged from six months to 13 years, but was less than two years in half of the cases. However, this uses a very conservative definition of the last time the donor was in an endemic area as the time of acquisition, thus all the estimates here are minimum possible estimates and very likely underestimates of true durations of infection. Donors included immigrants from malaria-endemic countries and non-immune donors who had acquired their malaria abroad, e.g., soldiers. The time between the transfusion and the onset of symptoms varied between four days and four weeks. This suggests a variety of inocula and multiplication rates (Figures 1 and 2A).

**Table 1:** **Transfusion or transplant transmitted falciparum malaria occurring at least six months after the donor’s last exposure risk**

Publication, country, reference	Description of recipient	Description of donor	Diagnostic test results for donor	Time lapse since donor’s last infection/risk of infection
Black *et al*., Australia [21]	Adult female with carcinoma; peri-operative blood transfusion in 1958, with fever 7 days post transfusion. Falciparum malaria diagnosed subsequently (confirmed by microscopy 14 days after transfusion)	Merchant seaman with trips to malarious areas 1942–7 and intermittent anti-malarials. History of malaria, most recent in Solomon Islands in 1953. Back in Australia since April 1957.	Slide positive	15 months
Chojnacki *et al*., USA [22]	60-years-old male with anaemia and myocardial infarction transfused who developed fever and chills 4 days post transfusion with falciparum malaria confirmed subsequently	19-years-old soldier, served in Vietnam until 7 months before blood donation. Had taken prophylaxis. Recalled short fever episode in Vietnam	>20 slides negative. Sternal bone marrow aspirate + by microscopy	7 months
Fisher *et al.*, USA [23]	Case 1: 54-years-old American female transfused during aorto-iliac bypass graft surgery in 1968. Developed fever 13 days post transfusion with falciparum malaria confirmed by microscopy nine days later. No relevant travel history	22-years-old American soldier in Vietnam 1966- March 1967. Took prophylaxis.	Positive serology; negative microscopy	13 months
	Case 2: 25-years-old female, transfused for post-partum haemorrhage. Developed fever ~2 weeks later and then severe malaria with coma. Diagnosis finally made 3 weeks post transfusion.	21-years-old American soldier in Viet Nam 1967–1968. Took prophylaxis.	Negative microscopy	7 months
Brooks *et al*.,USA[24]	56-years-old male with laryngeal carcinoma. Post-surgical transfusion; no relevant travel history. Died of cerebral malaria about 3 weeks post operatively.	Nigerian student.	Rare *P.F*T seen finally after multiple negative smears and a 500 ml phlebotomy.	32 months
Dike, UK [25]	Adult male, given blood transfusion after a road traffic accident. Developed fever 19 days later and falciparum malaria confirmed 24 days after last transfusion	Nigerian student. Asymptomatic	Serology positive.	17 months
Seligman *et al*., USA [26]	34-years-old male. End-stage renal failure on haemodialysis, born in Italy, in USA since 1955. Monthly blood transfusions. Developed falciparum malaria in 1970 with symptoms 7 days post transfusion. No relevant travel history.	Ghanaian, immigrated to USA in Oct 1969. No history of malaria.	One *P.F*T ring form found on thin smear. Serology positive.	13 months
Duizabo *et al*., France [27]	No details^*^	No details	No details	16 months
Besson *et al.,* France [28]	30-years-old male. Orthopaedic surgical procedure (correction of pseudarthrosis) with transfusion in 1973. Fever 2 weeks later.Eventual microscopy diagnosis of falciparum malaria. Had never left France and no history of malaria.	Donor born in Senegal, living in France since 1960, no history malaria.	Serology (IF) positive	13 years
Saleun *et al.,* France [29]	No details. *P. falciparum* transmitted by blood transfusion	Donor from Burkina Faso	No details	16 months
Duperval *et al.,* Canada [30]	8-years-old Canadian male, treated for chronic lymphocytic leukaemia, admission with high fever. Cerebral malaria diagnosed *post-mortem*, including sequestration of parasitised erythrocytes in cerebral vasculature. Last recorded blood transfusion was post gastrectomy 12 years earlier. No travel; route/time of malaria acquisition unknown. 50% parasitaemia on *pre-mortem* slides examined retrospectively.	No donor history	No details	12 years
Babinet *et al*., France [31]	53-years-old Portuguese female with left ventricular insufficiency, admitted for heart transplant. Developed symptoms of malaria 12 days post op, diagnosed on day 18. She died 4 days later^**^	51-years-old female originally from Cameroon	Donor's serum tested retrospectively was positive for *P. falciparum* Abs and Ag	15 months
Yarrish *et al*., USA [32]	65-years-old female, transfused at cardiothoracic surgery (for rheumatic mitral stenosis) in 1982. Developed post transfusion falciparum malaria with 5% parasitaemia diagnosed by microscopy 16 days post surgery. No relevant travel history.	Ghanaian male residing in USA for 6 years. Last trip to Ghana the year prior to donation	Positive *P.F* serology	10 months
Stickland *et al.,* Australia [33]	83-years-old female with myelodysplastic syndrome; transfusion dependent. 1991: developed falciparum malaria 23 days post- transfusion diagnosed by microscopy of blood and bone marrow aspirate. No relevant travel history.	Donor was previously a long-term resident of Papua New Guinea.	No laboratory confirmation	8 months
Slinger *et al.,* Canada [34]	Case 1: 62-years-old female, post transfusion falciparum malaria in 1997. No history of travel to malarious area	Donor 1: 19-years-old female from Ghana originally and living in Canada for 4 years.	Unable to get a sample for screening	4 years
	Case 2: 24-years-old female developed fever 15 days post transfusion with *P. falciparum* confirmed 3 days later	Donor 2: male, originally from Mali. History of malaria treated with chloroquine in 1991	Donor 2: PCR positive for *P.F*	4 years
	Case 3: 63-years-old male, *P. falciparum* malaria 16 days after platelet transfusion	Donor 3: Originally from Cameroon.	Donor 3: Rare *P.f.*T and gametocytes slide	3 years and history of malaria 13 years previously
Frey-Wettstein *et al*., Switzerland [35]	70-years-old male with ischaemic heart disease and aortic aneurysm, transfused peri-operatively in 1999. Developed post-transfusion falciparum malaria with symptoms 2 weeks post-op (confirmed by microscopy 1 week later) and died.	30-years-old male born in Cameroon, with history of malaria aged 15 years. Moved to Europe 10 years before blood donation. Last trip to Cameroon 6 years before.	Serology positive and later slide positive (gametocytes and *P.f.*T)	6 years
MMWR USA [36]	69-years-old male, transfused in 2003 following upper gastrointestinal bleed. No relevant travel history. Re-admitted 2–3 weeks post transfusion with falciparum malaria diagnosed by microscopy.	18-years-old blood donor of Ghanaian origin living in USA for 11 months. Treated for malaria 2 years previously.	Slide and PCR negative. Serology (IFA) positive.	11 months
Bruneel *et al.,* France [37]	81-years-old male, Type 2 diabetes mellitus, post transfusion cerebral malaria and death in 2002. Symptoms developed 2 weeks post transfusion. No travel to an endemic area for 20 years.	19-years-old female, originally from sub-Saharan Africa. Living in France for 4 years	Positive PCR for P.falciparum and negative thick film' in the 'Diagnostic test results for donor'	4 years
Kitchen, UK [38]	Case 1: 72-years-old female with acute leukaemia, never visited malarious area. Developed falciparum malaria 13 days after multiple blood product transfusions (probably from platelet transfusion)	Male, UK born, worked in Africa for 10 years and history of malaria.	Serology (IFAT) positive	2 years
	Case 2: Male with diabetes and Non-Hodgkins Lymphoma, presenting with falciparum malaria more than 2 weeks after a transfusion. Travelled to Kenya the year before	Female, Nigerian but UK resident since childhood. Three trips to Ghana	Serology (EIA and IFAT) positive	6 months
	Case 3: 62-years-old male, fever 4 days post transfusion for gastrointestinal bleeding in 1997. Served in Egypt in WWII. Otherwise no travel. Died of cerebral malaria.	19-years-old, Ghanaian female. UK national since aged 6 months. Travelled to Uganda in 1994 travelled to Uganda for 6 weeks where she reported undiagnosed fever.	Slide negative. Serology (EIA and IFAT) positive	3 years
	Case 4: 51-years-old male with sickle cell anaemia and chronic renal failure. Post transfusion falciparum malaria in 2003. No history of travel to malarious area. Left Jamaica for UK in 1957.	38-years-old female, Ghanaian, visited Ghana last in 1996	Slide positive Serology (EIA and IFAT) positive	7 years
MMWR USA [39]	25-years-old female with sickle cell anaemia, multiple transfusions in 2007, admitted with falciparum malaria (16% parasitaemia) more than 1 month post transfusion.	Donor of Nigerian origin in USA since 2001 with no travel in the intervening period. Hospitalized with fever presumed malaria in 1988	Serology positive	6 years
MMWR USA [40]	Case 1: 27-years-old male with fever 3 weeks post blood transfusion. No travel outside Florida	27-years-old old donor of Nigerian origin in USA since 2004 with no travel in the intervening period. History of malaria 15 years previously	Serology (IFA) positive, PCR negative	5 years
	Case 2: 78-years-old male receiving chemotherapy for lung carcinoma diagnosed with falciparum malaria after anaemia investigated. Several blood transfusions in previous 10 months, most recent 2–4 weeks previously. No travel in the last year.	30-years-old female, lived in East Africa as a child and travelled to Uganda, China, Brazil 13–17 months before donating	PCR negative, Serology (IFA) positive	13-17 months
MMWR USA [41]	55-years-old female; cardiac surgery and blood transfusion. Re-admitted 1 month later with falciparum malaria (microscopy and PCR positive). No recent relevant travel.	21-years-old male, born in Russia, lived in Benin for 17 years before immigrating to USA. No travel to endemic area in past 4 years	Slide and RDT neg, Serology and PCR positive (and PCR matched recipient *P.F*)	4 years
Lefavour *et al*., USA [42]	27-years-old Ghanaian female, living in USA since 1972 with end stage renal failure. Developed post-transplant falciparum malaria 2 weeks after receiving live related transplant from her mother. Treated, rejected kidney, graft nephrectomy. Second transplant a year later (cadaveric). Post transplant falciparum malaria 5 months later.	Donor 1 (mother)- from Ghana	Donor 1 (mother): serology positive	23 months
		Donor 2: no information	Donor 2: serology negative	

*P.f*.T: *P. falciparum* trophozoites, *: abstract only, **: Kidneys also transplanted to 2 recipients, 1 died of CMV but seroconverted for malaria, the other did not seroconvert.

Table 2 and Figure 2 summarize 32 cases of falciparum malaria in individuals diagnosed between seven months and 15 years after leaving a malaria endemic area. *Plasmodium falciparum* gametocytaemia was reported in three of the cases. In one case, gametocytes were the only parasite stage seen on the blood film a month after a fever episode, suggesting the patient had self-cured the infection. Five cases reported having taken antimalarial drugs previously. In 13 cases the symptomatic episode coincided with pregnancy (seven women) or was associated with a co-morbidity, e.g., diagnosis of malignancy (one), diabetes mellitus (one), post head-injury (one), sickle-cell anaemia (one), HIV (two cases), which suggests a reduction in host immunity may have unmasked the latent infection.

**Table 2:** **Non-transfusion cases of delayed presentation of falciparum malaria**

Author, country, reference	Case description	Time interval since last infection or exposure
Nagley *et al.*, UK [43]	49-years-old male, British soldier, in India from 1919 to 1928. History of malaria in India and one unconfirmed “relapse”. Self-treated with quinine in 1932. No other relevant travel, or blood transfusions. Developed jaundice and then severe malaria in 1945. *P. falciparum* trophozoites and gametocytes on microscopy	13-17 years
Walters *et al*., UK [44]	21-years-old pregnant female, originally from Nigeria. Asymptomatic. Diagnosis made by microscopy performed to investigate abnormal routine antenatal full blood count in 1958	19 months
Russell *et al.,* USA [45]	Medical student with annual episodes of smear positive falciparum malaria for 4 years after return from the Belgian Congo in 1934. Eventually cleared following a prolonged course of quinine	4 years
Revel *et al.,* France [46]	7-years-old male, originally from Comoros islands presenting with a febrile illness. *P. falciparum* trophozoites and gametocytes seen on microscopy, mixed with *P. ovale*.	3 years
Kyronseppa *et al.*, Finland [47]	46-years-old male sailor, last travel to endemic area was Jeddah 20 months previously. Other possible occupational exposure in late 1970s. Presented with fever after head injury (intracerebral bleed). *P. falciparum* diagnosed by microscopy	20 months
Krajden *et al*., Canada [48]	30-years-old male, originally from Ghana. Presented with diabetic ketoacidosis. Malaria diagnosed by microscopy. Serology positive.	32 months
MMWR USA [49]	Obstetrician/gynaecologist admitted with fever. Last trip to a malarious area was 15 years before (Afghanistan). Diagnosed on microscopy. Labelled as cryptic malaria (occupational exposure cannot be excluded).	15 years (unless occupational exposure)
Howden *et al.,* Australia [16]	28-years-old female, born in Eritrea and then lived in Sudan. Post splenectomy malaria. Pre-operative slides negative. Retrospective histological diagnosis of hyperreactive malarial splenomegaly.	9 years
Giobbia *et al.,* Italy [50]	29-years-old pregnant woman. Originally from Ghana but living in Italy for 9 years. Last trip to Ghana 4 years previously. Presented with fever and vomiting. Positive *P. falciparum* rapid diagnostic test, microscopy and serology.	4 years
Lesko *et al*., USA [51]	Congenital falciparum malaria in the infant of a mother who had not been in Haiti for 7 years	7 years
Greenwood *et al.*, Sweden [52]	18-years-old male with sickle-cell anaemia, born in Togo, living in Sweden for 14 years. Last visit 4 years previously. Presented with fever. Microscopy, RDT and PCR positive for *P. falciparum*.	4 years
D’Ortenzio *et al.*, France [53]	11 cases summarized as part of a case–control study (variable level of detail):	
	26-years-old male, born in France and lived in Madagascar for 2 years where he took regular chloroquine + primaquine prophylaxis. Presented with severe malaria 7 months after return	7 months
	5 cases (2 HIV+) with >1 year time lapse since arrival in France	>1 year
	5 pregnant women	>3 years
Theunissen *et al.*, Belgium [54]	30-years-old male, originally from Guinea-Conakry and moved to Belgium 9 years earlier. No other travel of note. Friend from Guinea stayed with him 2 weeks earlier (raises possibility of ‘baggage malaria’).	9 years
Foca *et al.,* Italy [55]	60-years-old Italian engineer, worked in Tanzania for 33 years where he was treated for malaria. Returned to Italy January 2006 and was diagnosed with carcinoma of the lung and splenomegaly. Fever episode 11 months after return to Italy which resolved spontaneously and *P. falciparum* gametocytes seen on a blood smear 1 month later.	11 months
Szmitko *et al.,* Canada [56]	29-years-old Angolan female. Immigrated to Canada in 1999, no relevant travel in intervening period. History of treated malaria in Angola. Presented with fever. Falciparum malaria diagnosed by microscopy and PCR.	8 years
MMWR USA [41]	Case 1: 17-years-old female, USA born, Ghanaian mother, last travel 4 years previously (report implies to Ghana but not stated explicity). Admitted to hospital with falciparum malaria. No details on diagnosis	4 years
	Case 2: 31-years-old female, presented to the emergency department with severe falciparum malaria (10% parasitaemia). Travelled to Nicaragua in 2008; took prophylaxis.	2 years
MMWR USA [57]	8-years-old female, originally from Nigeria. Moved to USA 2007, one trip back in 2008. Malaria history not documented. Presented symptomatic to USA hospital. *P. falciparum* diagnosis confirmed by microscopy and PCR.	3 years
Monge-Maillo *et al.,* Spain [58]	3 cases: No details on clinical presentation or diagnosis. Only age, sex and country of probably acquisition	
	Case 1 : 32 -years-old male, Angola	13 months
	Case 2 : 17-years-old male, Senegal	14 months
	Case 3 : 28-years-old male, Guinea	28 months
Berrevoets *et al.*, Netherlands [59]	48-years-old male, born in Burkina Faso, immigrated to Netherlands 8 years previously, last travel to an endemic country 2.5 years previously. Last anti-malarial drugs in 1997. Admitted with severe malaria confirmed by RDT, microscopy (*P. falciparum* trophozoites and schizonts) and PCR.	2.5 years

RDT: rapid diagnostic test, PCR: polymerase chain reaction, IFA: indirect fluorescent antibody assay.

### Detection of occult malaria infections

The ability to detect asymptomatic carriers of malaria parasites depends on the sensitivity of the detection method used. Blood smear microscopy by experienced microscopists will detect parasite densities of ≥50/μL reliably. More recently, molecular methods have been employed to detect sub-patent parasitaemias. PCR is considerably more sensitive than microscopy or rapid diagnostic tests based on antigen detection but typically blood samples have been taken from finger-prick blood spots on filter paper, thus a volume of only ~5 μL has been amplified. This obviously misses densities which are below one parasite in 5 μL or 200 parasites/mL. In general, most PCR methods have limits of detection of ≥1,000 parasite/mL. Using larger blood volumes parasite densities down to ~20/mL can be detected, several orders of magnitude below the microscopy level of detection [60].

Malaria infection may also be assessed using serology, although interpretation is difficult. A variety of crude and more refined test antigens have been evaluated. Typically seroprevalence increases with age while the correlation with patent parasitaemia goes down [61, 62]. The usual duration of positive malaria antibody responses once an infection has been cleared with or without anti-malarials, in the absence of ongoing exposure, and the specificity of negative serology results to exclude active infection are both uncertain.

Longitudinal serological studies have been conducted in immigrants who have moved to countries that are malaria free. In a Spanish study IgG antibody levels against the erythrocytic antigens AMA-1 and MSP-142 (3D7 and FVO strains), EBA-175 and DBL-α, did not correlate with the time since migration [63]. Serology is used by a number of transfusion services for blood donor screening. Between 2005 and 2011, in the USA, 5,610 malaria-deferred donors were tested by EIA, including 5,412 travel deferrals. Overall, 88 (1.6%) of tests were EIA reactive on repeat testing, none of which were PCR positive [64]. In the transfusion-transmitted malaria cases summarized in Table 1, 11 donors had positive serology between ten months and 13 years after leaving an endemic area but were PCR or slide negative, suggesting that isolated positive serology may signify persistent low density parasitaemia below the detection threshold of conventional (i.e., low blood volume) PCR techniques which is approximately 1 parasite/μL. Given these uncertainties many blood transfusion services opt for a conservative policy of excluding donors who have lived in malaria-endemic areas.

### Transmissibility of sub-microscopic *Plasmodium falciparum*infections

There is good evidence that chronic sub-patent infections are a source of onward mosquito transmission of malaria. In semi-immune individuals with persistent infections, gametocytaemia at densities below the limit of microscopy detection is often infectious [14]. In 1931 in South Africa, Swellengrebel estimated that crescent densities of only 1: 5,100 leucocytes were sufficient to infect anopheline mosquitoes and in 1945 Robertson recorded infections at thresholds below three crescents per 1,000 leucocytes in *Anopheles gambiae* in West Africa [5]. Ciuca *et al.* studied the infectivity of long-term asymptomatic *P. falciparum* malaria to laboratory-bred *Anopheles atroparvus* following experimentally induced infections in ten individuals [6]. They observed infectivity rates of 0.7-7% even when no gametocytes were seen on microscopy examination of three thick films. Transmission was confirmed up to the 175^th^ day after infection.

## Conclusions

These reports show unequivocally that *P. falciparum* can survive for extended periods in the human host. The previously quoted maximum duration of two years should be revised; 13 years is the maximum confirmed duration reported to date. The transfusion malaria cases suggest the parasite continues its erythrocytic life cycle at very low densities, kept in check by the host’s immune response. This is a common pattern for malaria parasites in all animals, and is the most likely explanation for persistence of both *P. falciparum* and *P. malariae* in man. There is no evidence for protracted dormancy or an exo-erythrocytic parasite stage of these parasites, although it is possible that the entire life cycle could take place in the vascular spaces in the placenta or spleen. Incomplete antimalarial drug treatment may have a modulatory effect on the infection early on. As well as the poorly understood host’s response to the parasite, *P. falciparum* possesses sophisticated immune evasion mechanisms, notably the differential serial expression of *var* genes encoding for *Pf*EMP1, the major surface antigen of the blood stage [65]. How *P. falciparum* parasites survive for prolonged periods in the human host without exhausting their repertoire of antigenic variation is unclear. The appearance of symptoms years after infection may reflect waning of host immunity. The late recrudescing infections presented in Table 2 were associated with concomitant conditions, which may have led to immunosuppression in several cases, e.g., diabetes mellitus, malignancy, and pregnancy.

It is not possible to estimate how common chronic infection with *P. falciparum* might be. The World Malaria Report estimated that in 2012 there were between 135 and 287 million malaria cases of which 91% were caused by *P. falciparum* [66]. Even if only a tiny proportion of untreated or partially treated infections become indolent, this represents a huge potential reservoir of sustained transmission.

Approximately 107 million units of blood donations are collected globally every year [67]. Very few blood transfusion services perform routine malaria antibody testing, with most non-endemic countries relying on deferral of donors considered to be at risk. This appears to be very effective in preventing the vast majority of cases of transfusion-transmitted malaria [68]. It is also possible that some transfusion-transmitted parasites do not cause disease. A large proportion of the transfusion-recipients presented in Table 1 were older, with co-morbidities such as malignancy, diabetes, end-stage renal failure, and sickle-cell anaemia and thus may have been more susceptible to malaria following inoculation of very low numbers of parasites than immunocompetent recipients. The requirement for blood transfusion indicates anaemia and therefore illness. Furthermore, blood transfusion itself is associated with immunosuppression [69]. The incidence of transfusion-transmitted malaria in endemic countries is probably far higher than realised since screening by microscopy only is usually the method used to select donors and antimalarial drugs are not added to blood donations. Transfusion malaria could become an important source of malaria within countries where there is incomplete elimination.

These observations on the longevity of falciparum infections and the sub-microscopic parasite burden are relevant to immigrants and refugees who have left malarious areas. In a parasite prevalence study of 195 African refugees in Italy in 2007, 62 patients tested positive for malaria using molecular detection methods, of whom 13 were gametocytaemic. Microscopy confirmed the positive results in 14 of the 62 cases, of which 13 were *P. falciparum* [70]. The reported prevalence of asymptomatic malaria in refugees screened post-arrival ranges from 2.4 to 31.8%, depending on the population and the method of diagnosis. Where malaria vectors are present these people may act as a source of transmission. The possibility that asymptomatic infections with artemisinin-resistant falciparum malaria could be imported from Southeast Asia to Africa is a current concern.

In countries targeting malaria elimination, achieving this aim is likely to take longer if the sub-microscopic parasites are not eliminated. Improved high-throughput and low-cost methods are needed to detect these individuals harbouring low parasite densities. Conventional PCR methods still miss infections with densities below 1/uL. Serology has been proposed as a useful tool to detect changes in malaria transmission in settings where transmission is in decline and appears to be a sensitive method for the detection of malaria infection but its specificity is unclear, as is the usual duration of detectable antibodies once an infection has cleared. There is a need to investigate the role of serology in defining asymptomatic carriage, certainly in lower transmission and pre-elimination countries.

*Plasmodium falciparum* can persist in the human host for longer than thought previously. Current policies in malaria-free countries of excluding blood donors who have lived in malarious areas are justified. Vigilance for longer than three years after declaring elimination in an area may be needed.

## References

[1] Collins WE, Jeffery GM (2007). *Plasmodium malariae*: parasite and disease. Clin Microbiol Rev.

[2] Brouwer EE, van Hellemond JJ, van Genderen PJ, Slot E, van Lieshout L, Visser LG, Wismans PJ (2013). A case report of transfusion-transmitted *Plasmodium malariae* from an asymptomatic non-immune traveller. Malar J.

[3] Jeffery GM, Eyles DE (1954). The duration in the human host of infections with a Panama strain of *Plasmodium falciparum*. Am J Trop Med Hyg.

[4] Eyles DE, Young MD (1951). The duration of untreated or inadequately treated *Plasmodium falciparum* infections in the human host. J Natl Malar Soc.

[5] Covell G (1960). Relationship between malarial parasitaemia and symptoms of the disease: a review of the literature. Bull World Health Organ.

[6] Ciuca M, Lupasco G, Ballif-Negulici E, Constantinesco P, Cristesco A, Sandesco I (1963). Experimental research on the infectivity for A. atroparvus of P. vivax or P. falciparum asymptomatic parasitaemias in relation to acquired immunity in countries where malaria is endemic.

[7] Bruce-Chwatt LJ (1959). Malaria research and eradication in the USSR. A review of Soviet achievements in the field of malariology. Bull World Health Organ.

[8] Verdrager J (1964). Observations on the longevity of *Plasmodium falciparum*: with special reference to findings in Mauritius. Bull World Health Organ.

[9] Earle WC (1962). The Course of Naturally Acquired Malaria.

[10] Sama W, Killeen G, Smith T (2004). Estimating the duration of *Plasmodium falciparum* infection from trials of indoor residual spraying. Am J Trop Med Hyg.

[11] Hamad AA, El Hassan IM, El Khalifa AA, Ahmed GI, Abdelrahim SA, Theander TG, Arnot DE (2000). Chronic *Plasmodium falciparum* infections in an area of low intensity malaria transmission in the Sudan. Parasitology.

[12] al-Yaman F, Genton B, Reeder JC, Anders RF, Smith T, Alpers MP (1997). Reduced risk of clinical malaria in children infected with multiple clones of *Plasmodium falciparum* in a highly endemic area: a prospective community study. Trans R Soc Trop Med Hyg.

[13] Bruce MC, Galinski MR, Barnwell JW, Donnelly CA, Walmsley M, Alpers MP, Walliker D, Day KP (2000). Genetic diversity and dynamics of *Plasmodium falciparum* and *P. vivax* populations in multiply infected children with asymptomatic malaria infections in Papua New Guinea. Parasitology.

[14] Bousema T, Drakeley C (2011). Epidemiology and infectivity of *Plasmodium falciparum* and *Plasmodium vivax* gametocytes in relation to malaria control and elimination. Clin Microbiol Rev.

[15] Bidegain F, Berry A, Alvarez M, Verhille O, Huguet F, Brousset P, Pris J, Marchou B, Magnaval JF (2005). Acute *Plasmodium falciparum* malaria following splenectomy for suspected lymphoma in 2 patients. Clin Infect Dis.

[16] Howden BP, Vaddadi G, Manitta J, Grayson ML (2005). Chronic falciparum malaria causing massive splenomegaly 9 years after leaving an endemic area. Med J Aust.

[17] Woolsey G (1911). Transfusion for pernicious anemia: Two cases. Ann Surg.

[18] Fairley NH (1947). Sidelights on malaria in man obtained by subinoculation experiments. Trans R Soc Trop Med Hyg.

[19] Bruce-Chwatt LJ (1974). Transfusion malaria. Bull World Health Organ.

[20] Website of the International Haemovigilance network. [http://www.ihn-org.com/], **Website of the International Haemovigilance network.** [http://www.ihn-org.com/]

[21] Black RH (1960). Investigation of blood donors in accidental transfusion malaria-*Plasmodium vivax*, *falciparum* and *malariae* infections. Med J Aust.

[22] Chojnacki RE, Brazinsky JH, Barrett O (1968). Transfusion-introduced falciparum malaria. N Engl J Med.

[23] Fisher GU, Schultz MG (1969). Unusual host-parasite relationship in blood-donors responsible for transfusion-induced falciparum malaria. Lancet.

[24] Brooks MH, Barry KG (1969). Fatal transfusion malaria. Blood.

[25] Dike A, Draper CC (1970). A case of transfusion malaria due to *Plasmodium falciparum* with antibody studies of the donors. Trans R Soc Trop Med Hyg.

[26] Seligman SJ, Choa MS (1971). Transfusion-induced falciparum malaria. JAMA.

[27] Duizabo P, Romano P, Usdin JP, Barbizet J (1974). [Transfusion induced malaria with serious mental disturbance. Report of a case](in French). Concours Med.

[28] Besson P, Robert JF, Reviron J, Richard-Lenoble D, Gentilini M (1976). A propos de deux observations de paludisme transfusionnel. Essai de prevention associant un test d'immunofluorescence indirecte aux criteres de selection clinique. Revue Francaise de Transfusion et Immuno-hematologie.

[29] Saleun JP, Baret M, Dauriac J (1976). Resultats d'une enquire sur le paludisme post-transfusionnel en France entre 1960 et 1974. Revue Francaise de Transfusion et Immuno-hematologie.

[30] Duperval R, Longpre B, Madarnas P (1979). Unexplained falciparum malaria in a patient with chronic lymphocytic leukemia. Can Med Assoc J.

[31] Babinet J, Gay F, Bustos D, Dubarry M, Jaulmes D, Nguyen L, Gentilini M (1991). Transmission of Plasmodium falciparum by heart transplant. BMJ.

[32] Yarrish RL, Janas JS, Nosanchuk JS, Steigbigel RT, Nusbacher J (1982). Transfusion malaria: treatment with exchange transfusion after delayed diagnosis. Arch Intern Med.

[33] Stickland JF, Roberts AN, Williams V (1992). Transfusion-induced malaria in Victoria. Med J Aust.

[34] Slinger R, Giulivi A, Bodie-Collins M, Hindieh F, John RS, Sher G, Goldman M, Ricketts M, Kain KC (2001). Transfusion-transmitted malaria in Canada. Can Med Assoc J.

[35] Frey-Wettstein A, Maier M, Markwalder K, Münch U (2001). A case of transfusion transmitted malaria in Switzerland. Swiss Med Wkly.

[36] Eliades MJ, Shah S, Nguyen-Dinh P, Newman RD, Barber AM, Nguyen-Dinh P, Roberts JM, Mali S, Parise ME, Barber AM, Steketee R (2005). Malaria surveillance–United States, 2003. MMWR Surveill Summ.

[37] Bruneel F, Thellier M, Eloy O, Mazier D, Boulard G, Danis M, Bedos JP (2004). Transfusion-transmitted malaria. Intensive Care Med.

[38] Kitchen AD, Barbara JA, Hewitt PE (2005). Documented cases of post-transfusion malaria occurring in England: a review in relation to current and proposed donor-selection guidelines. Vox Sang.

[39] Centers for Disease C, Prevention (2010). Travel-associated Dengue surveillance - United States, 2006–2008. MMWR Morb Mortal Wkly Rep.

[40] Mali S, Tan KR, Arguin PM (2011). Division of Parasitic Diseases, Malaria CfGH, Centers for Disease Control and Prevention: Malaria surveillance–United States, 2009. MMWR Surveill Summ.

[41] Mali S, Kachur SP, Arguin PM (2012). Division of Parasitic Diseases, Malaria CfGH, Centers for Disease Control and Prevention: Malaria surveillance–United States, 2010. MMWR Surveill Summ.

[42] Lefavour GS, Pierce JC, Frame JD (1980). Renal transplant-associated malaria. JAMA.

[43] Nagley L (1945). Probable relapse of malignant tertian malaria after thirteen years. Lancet.

[44] Walters J (1960). Quiescent malarial parasites. BMJ.

[45] Russell PF, West LS, Manwell RD, Macdonald G (1963). Practical Malariology.

[46] Revel MP, Datry A, Saint Raimond A, Lenoir G, Danis M, Gentilini M (1988). *Plasmodium falciparum* malaria after three years in a non-endemic area. Trans R Soc Trop Med Hyg.

[47] Kyrönseppä H, Tiula E, Repo H, Lähdevirta J (1989). Diagnosis of falciparum malaria delayed by long incubation period and misleading presenting symptoms: life-saving role of manual leucocyte differential count. Scand J Infect Dis.

[48] Krajden SP, Panisko DM, Tobe B, Yang J, Keystone JS (1991). Prolonged infection with *Plasmodium falciparum* in a semi-immune patient. Trans R Soc Trop Med Hyg.

[49] Shah S, Filler S, Causer LM, Rowe AK, Bloland PB, Barber AM, Roberts JM, Desai MR, Parise ME, Steketee RW (2004). Malaria surveillance–United States, 2002. MMWR Surveill Summ.

[50] Giobbia M, Tonon E, Zanatta A, Cesaris L, Vaglia A, Bisoffi Z (2005). Late recrudescence of *Plasmodium falciparum* malaria in a pregnant woman: a case report. Int J Infect Dis.

[51] Lesko CR, Arguin PM, Newman RD (2007). Congenital malaria in the United States: a review of cases from 1966 to 2005. Arch Pediatr Adolesc Med.

[52] Greenwood T, Vikerfors T, Sjoberg M, Skeppner G, Farnert A (2008). Febrile *Plasmodium falciparum* malaria 4 years after exposure in a man with sickle cell disease. Clin Infect Dis.

[53] D'Ortenzio E, Godineau N, Fontanet A, Houze S, Bouchaud O, Matheron S, Le Bras J (2008). Prolonged *Plasmodium falciparum* infection in immigrants, Paris. Emerg Infect Dis.

[54] Theunissen C, Janssens P, Demulder A, Nouboussie D, Van-Esbroeck M, Van-Gompel A, Van-Denende J (2009). Falciparum malaria in patient 9 years after leaving malaria-endemic area. Emerg Infect Dis.

[55] Foca E, Zulli R, Buelli F, De Vecchi M, Regazzoli A, Castelli F (2009). *P. falciparum* malaria recrudescence in a cancer patient. Infez Med.

[56] Szmitko PE, Kohn ML, Simor AE (2009). *Plasmodium falciparum* malaria occurring 8 years after leaving an endemic area. Diagn Microbiol Infect Dis.

[57] Cullen KA, Arguin PM (2013). Division of Parasitic Diseases, Malaria CfGH Centers for Disease Control and Prevention: Malaria surveillance–United States, 2011. MMWR Surveill Summ.

[58] Monge-Maillo BN, Norman F, Pérez-Molina JA, Díaz-Menéndez M, Rubio JM, López-Vélez R (2012). *Plasmodium falciparum* in asymptomatic immigrants from sub-Saharan Africa, Spain. Emerg Infect Dis.

[59] Berrevoets MA, Sprong T, Meis JF, Dofferhoff AS (2013). *Plasmodium falciparum* malaria recrudescence occurring 2.5 years after leaving an endemic country. Neth J Med.

[60] Imwong M, Hanchana S, Malleret B, Renia L, Day NP, Dondorp A, Nosten F, Snounou G, White NJ (2014). High throughput ultra-sensitive molecular techniques to quantify low density malaria parasitaemias. J Clin Microbiol.

[61] Satoguina J, Walther B, Drakeley C, Nwakanma D, Oriero EC, Correa S, Corran P, Conway DJ, Walther M (2009). Comparison of surveillance methods applied to a situation of low malaria prevalence at rural sites in The Gambia and Guinea Bissau. Malar J.

[62] Hsiang MS, Hwang J, Kunene S, Drakeley C, Kandula D, Novotny J, Parizo J, Jensen T, Tong M, Kemere J, Dlamini S, Moonen B, Angov E, Dutta S, Ockenhouse C, Dorsey G, Greenhouse B (2012). Surveillance for malaria elimination in Swaziland: a national cross-sectional study using pooled PCR and serology. PLoS One.

[63] Moncunill G, Mayor A, Bardaji A, Puyol L, Nhabomba A, Barrios D, Aguilar R, Pinazo MJ, Almirall M, Soler C, Dlamini S, Moonen B, Angov E, Dutta S, Ockenhouse C, Dorsey G, Greenhouse B (2013). Cytokine profiling in immigrants with clinical malaria after extended periods of interrupted exposure to *Plasmodium falciparum*. PLoS One.

[64] Nguyen ML, Goff T, Gibble J, Steele WR, Leiby DA (2013). Analyzing actual risk in malaria-deferred donors through selective serologic testing. Transfusion.

[65] Barry AE, Leliwa-Sytek A, Tavul L, Imrie H, Migot-Nabias F, Brown SM, McVean GA, Day KP (2007). Population genomics of the immune evasion (var) genes of *Plasmodium falciparum*. PLoS Pathog.

[66] WHO (2013). World Malaria Report 2013.

[67] World Health Organisation: **10 facts on blood transfusion.** http://www.who.int/features/factfiles/blood_transfusion/en/]

[68] Reesink HW, Panzer S, Wendel S, Levi JE, Ullum H, Ekblom-Kullberg S, Seifried E, Schmidt M, Shinar E, Prati D, Berzuini A, Ghosh S, Flesland Ø, Jeansson S, Zhiburt E, Piron M, Sauleda S, Ekermo B, Eglin R, Kitchen A, Dodd RY, Leiby DA, Katz LM, Kleinman S (2010). The use of malaria antibody tests in the prevention of transfusion-transmitted malaria. Vox Sang.

[69] Vamvakas EC, Blajchman MA (2007). Transfusion-related immunomodulation (TRIM): an update. Blood Rev.

[70] Marangi M, Di Tullio R, Mens PF, Martinelli D, Fazio V, Angarano G, Schallig HD, Giangaspero A, Scotto G (2009). Prevalence of Plasmodium spp. in malaria asymptomatic African migrants assessed by nucleic acid sequence based amplification. Malar J.

